# Prolonged fever as a harbinger: the diagnostic and therapeutic challenge of recurrent complicated urinary tract infection following congenital dermal sinus excision in an infant: a case report

**DOI:** 10.3389/fped.2026.1900708

**Published:** 2026-10-21

**Authors:** Lihui Liao, Wentao Peng

**Affiliations:** 1 WCSUH-Tianfu Sichuan Provincial Children’s Hospital (Sichuan Provincial Children’s Medical Center), Chengdu, China; 2 Department of Nursing, West China Second University Hospital, Sichuan University, Chengdu, China; 3 Key Laboratory of Birth Defects and Related Diseases of Women and Children (Sichuan University), Ministry of Education, Chengdu, Sichuan, China

**Keywords:** Candida glabrata, congenital dermal sinus, Enterococcus faecium, infant, neurogenic bladder, refractory urinary tract infection, spinal epidermoid cyst

## Abstract

**Background:**

Congenital dermal sinus (CDS) is a rare form of occult spinal dysraphism. Although neurological deterioration is well recognized, postoperative neurogenic bladder and subsequent recurrent complicated urinary tract infection (rcUTI) in infants are less well described.

**Case presentation:**

A 9-month-and-26-day-old girl presented with 4 weeks of intermittent fever followed by acute paraplegia. MRI revealed a CDS with an infected intradural epidermoid cyst, which was resected. Postoperatively, she developed a neurogenic bladder requiring catheterization. Sequential urine cultures revealed Klebsiella aerogenes, fluconazole-resistant Candida glabrata (evolving from dose-dependent susceptibility), and multidrug-resistant Enterococcus faecium, accompanied by bladder stones and hydronephrosis. Blood cultures were negative on multiple occasions, with no bacterial growth for 5 consecutive days. A prolonged, susceptibility-guided antimicrobial regimen plus stone removal achieved sterilization. However, several positive urine cultures represented colonization rather than true infection; this is discussed as a cautionary antimicrobial-stewardship narrative.

**Conclusions:**

Three key conclusions emerged. First, prolonged fever without neurological signs may be an early indication of CDS. Second, neurogenic bladder following lumbosacral CDS can drive recurrent complicated UTI involving sequential bacterial and fungal pathogens. Third, successful rescue requires a multidisciplinary, prolonged, source-control-based strategy. This report adds to the limited literature by documenting detailed serial microbiological follow-up after CDS surgery. The emergence of fluconazole resistance in C. glabrata may have been promoted by treating colonization, highlighting the need for strict UTI definitions in catheterized infants.

## Introduction

Congenital dermal sinus (CDS) is a rare form of occult spinal dysraphism characterized by a persistent epithelial-lined tract from the skin surface to the spinal canal (1). It can serve as a direct conduit for central nervous system infection, leading to meningitis, intramedullary abscess, or infected spinal epidermoid cysts (1, 2). Infected epidermoid cysts may cause acute neurological deterioration, including rapid paraplegia, and usually require urgent neurosurgical resection (3, 4).

After lumbosacral CDS resection, some infants develop neurogenic bladder because of conus medullaris or cauda equina injury (5). Neurogenic bladder causes incomplete bladder emptying and often requires clean intermittent catheterization (CIC) (6), creating a high-risk environment for recurrent urinary tract infection (UTI) (3). In this setting, repeated instrumentation, residual urine, and biofilm formation can promote infection with multidrug-resistant (MDR) organisms (7, 8).

Recurrent complicated UTI is defined as persistent or recurrent complicated UTI despite appropriate therapy, often involving MDR pathogens (9, 10). It is mainly discussed in adult urology and is underrecognized in pediatrics (11, 12). The sequential microbiological evolution from bacterial to fungal pathogens after CDS surgery has rarely been detailed with serial susceptibility data in infants (13).

We report an infant with an infected spinal epidermoid cyst secondary to CDS who developed a neurogenic bladder and recurrent complicated UTI after neurosurgical resection. This case adds to the limited literature by providing serial urine cultures, susceptibility data, and a source-control-based salvage strategy.

## Case presentation

A 9-month-and-26-day-old female infant with a history of spina bifida occulta on prior MRI presented with 4 weeks of intermittent fever followed by acute flaccid paraplegia over 3 days. The fever was initially high (up to 39.6° C) and transiently responsive to antibiotics at a local hospital, but recurred (peak 38.6° C). On admission, vital signs were stable (T 37.7° C, HR 170 bpm, BP 80/49 mmHg). Neurological examination revealed flaccid paraplegia (0/5 muscle strength), complete sensory loss below the umbilicus, and areflexia. A sacral dimple with localized hypertrichosis was noted.

Admission laboratory tests revealed leukocytosis (WBC 12.6 × 10^9^/L) and elevated C-reactive protein (48.6 mg/L). Blood cultures were obtained before antimicrobial therapy and were negative on multiple occasions, with no bacterial growth for 5 consecutive days.

Emergency noncontrast MRI of the whole spine revealed diffuse spinal cord swelling and signal abnormality from the cervical to lumbar level. Contrast-enhanced MRI showed a linear tract from the sacral skin dimple extending into the spinal canal, consistent with CDS, and a non-enhancing intradural cystic mass at L1–S2. MRI also suggested a low-lying conus medullaris and tethered cord, which were confirmed intraoperatively. The mass was T1 hypointense/slightly hypointense and T2 heterogeneous hyperintense, with peripheral enhancement. Postoperative MRI showed residual lesion. MRI, intraoperative, and histopathological images were obtained at an outside hospital and could not be retrieved for de-identified publication.

On day 18 after admission, she underwent intraspinal lesion resection, including lumbar intraspinal lesion resection and drainage, dermal sinus excision, and spinal canal decompression. The dura was not actively opened, but a localized dural defect was found intraoperatively; the sinus tract entered the subdural space through this defect. There was no postoperative cerebrospinal fluid leak. The cystic lesion was completely resected, and the dermal sinus tract was ligated and divided. Histopathology confirmed an infected epidermoid cyst secondary to CDS. On 2025-11-07, she underwent “L1–S2 intraspinal giant lesion resection + tethered cord and filum terminale adhesion release + spinal nerve root decompression” at another hospital. Tethered cord and filum terminale adhesions were confirmed and released.

Purulent material from the cyst grew Klebsiella aerogenes (ceftriaxone-resistant, meropenem-susceptible). Postoperative serial urine cultures are summarized in Table 1. The clinical timeline is summarized in Table 2. The first positive urine culture was obtained on day 25 (approximately one week after surgery), before CIC was started. The patient developed a neurogenic bladder requiring CIC every 4–6 h. The following episodes were documented. The clinical timeline is shown in Figure 1, and the microbiological and antimicrobial timeline is shown in Figure 2:

Episode 1 (D25): Klebsiella aerogenes, >10^5 ^CFU/mL, collected by clean intermittent catheterization; urinalysis showed pyuria (WBC 3+), and the patient had fever (38.5° C). Treated as true infection with meropenem.
Episode 2 (D45): Candida glabrata, 6.8 × 10^4 ^CFU/mL, collected by catheterization; urinalysis showed pyuria and cloudy urine with sediment; the patient was afebrile but had persistent urinary sediment.
Episode 3 (D70–110): Enterococcus faecium, >10^5 ^CFU/mL (×4), collected by catheterization; urinalysis was normal on two occasions, and the patient was asymptomatic. This was considered colonization and was not treated with targeted therapy.
Episode 4 (Week 18–20): Candida glabrata, fluconazole-resistant (MIC 128), collected by catheterization; urinalysis showed pyuria. Treated with micafungin.

**Figure 1:**
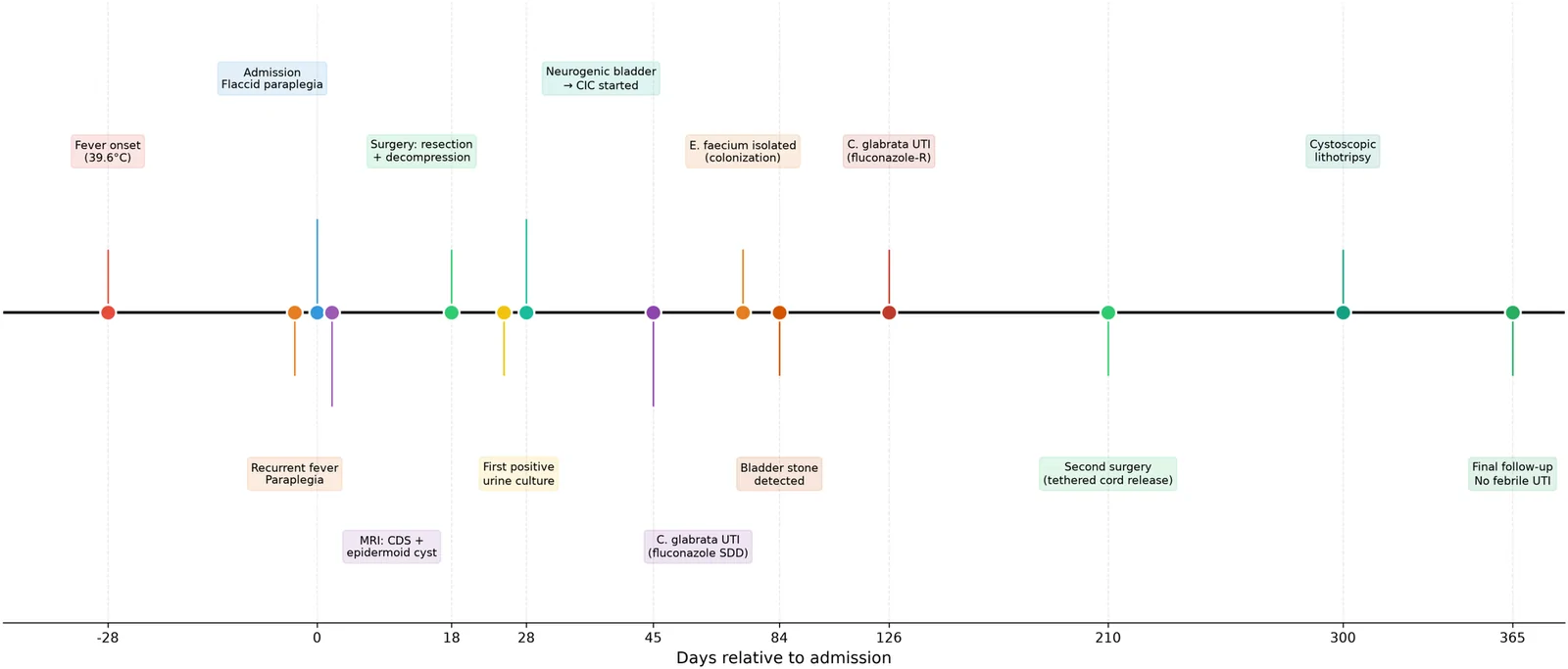
Clinical timeline of the case. Key events from fever onset to final follow-up are shown. UTI, urinary tract infection; CIC, clean intermittent catheterization.

**Figure 2:**
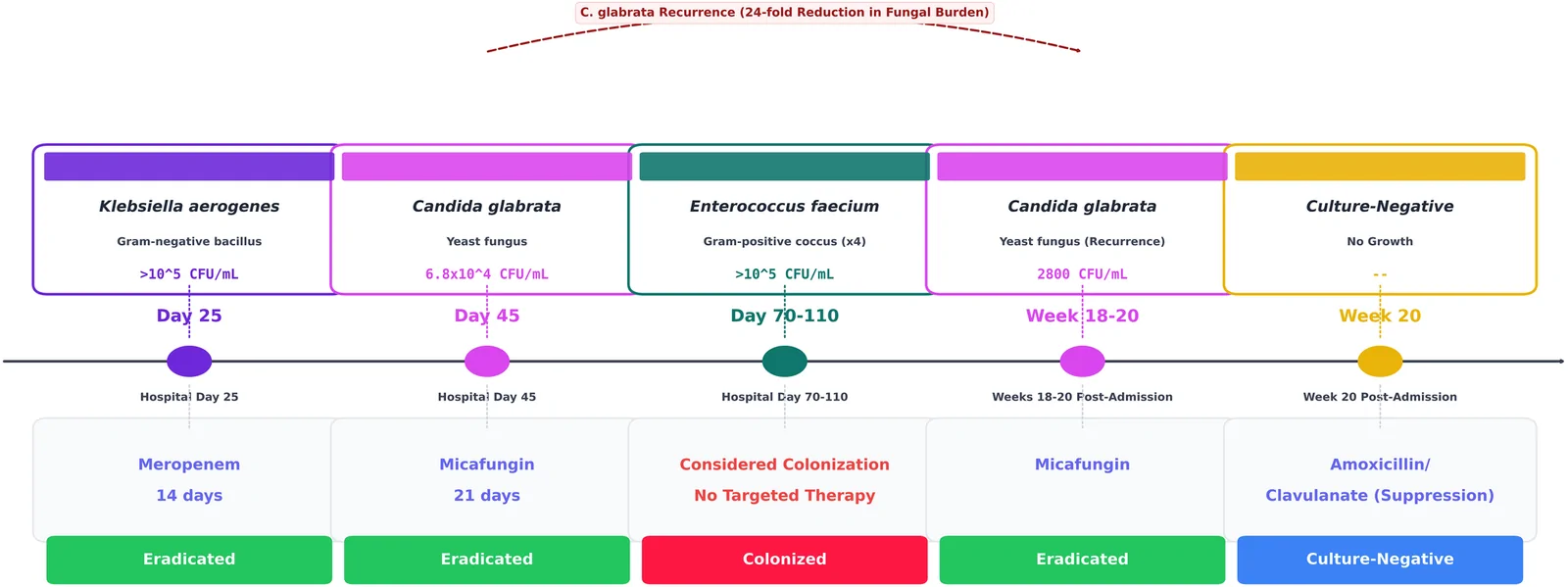
Microbiological and antimicrobial timeline. Sequential urine cultures and antimicrobial treatments are summarized. CFU, colony-forming units.

**Table 1:** Sequential urine culture results and antimicrobial therapy.

Time point	Pathogen	Colony count	Collection method	Pyuria	Clinical signs	Susceptibility	Therapy	Outcome
D25	Klebsiella aerogenes	>10^5^ CFU/mL	Catheterization	WBC 3+	Fever 38.5° C	Ceftriaxone R; meropenem S; cefoperazone-sulbactam S	Meropenem × 14 days	Cleared
D45	Candida glabrata	6.8 × 10^4^ CFU/mL	Catheterization	WBC 3+	Cloudy urine, sediment; afebrile	Fluconazole SDD; micafungin S; caspofungin I	Micafungin × 21 days	Cleared
D70–110	Enterococcus faecium	>10^5^ CFU/mL ( × 4)	Catheterization	Normal on two occasions	Asymptomatic	Ampicillin R; vancomycin S; linezolid R	Considered colonization; no targeted therapy	Colonization
Week 18–20	Candida glabrata	2,800 CFU/mL	Catheterization	WBC 3+	Urinary sediment	Fluconazole R; micafungin S; caspofungin I	Micafungin	Cleared

S, susceptible; R, resistant; SDD, susceptible dose dependent; I, intermediate.

**Table 2:** Clinical timeline.

Time	Event
Week 4	Fever onset
Week 0	Admission + acute paraplegia
D2	MRI diagnosis: CDS + epidermoid cyst
D18 (2025-08-28)	First surgery at our hospital: intraspinal lesion resection + dermal sinus excision + spinal canal decompression; dura not actively opened; no CSF leak
D25	First positive urine culture (Klebsiella aerogenes)—before CIC started
Week 3–4	Neurogenic bladder develops → CIC started
D45	Candida glabrata UTI
D70–110	Enterococcus faecium isolated—considered colonization
Week 12	Bladder stone detected
Week 18–20	Candida glabrata (fluconazole-resistant) UTI
2025-11-07	Second surgery at another hospital: L1–S2 intraspinal giant lesion resection + tethered cord and filum terminale adhesion release + spinal nerve root decompression
Week 20–24	Stone removal surgery (2026-02-10)
Week 24	Discharge, no further infections

We defined recurrent complicated UTI as ≥2 episodes of UTI with pyuria (>10 WBC/HPF) and clinical symptoms (fever or urinary symptoms), or a single episode with systemic involvement (14), in a patient with neurogenic bladder and CIC. Asymptomatic bacteriuria or candiduria without pyuria was not treated as infection.

For possible immune-mediated myelitis, she was empirically treated with intravenous ceftriaxone, vancomycin, methylprednisolone, and intravenous immunoglobulin. Postoperatively, she was switched to meropenem based on culture results. She developed a neurogenic bladder requiring CIC every 4–6 h. Residual urine was monitored by ultrasound, and a bladder diary was kept. Because of recurrent UTI, temporary indwelling catheterization and intravenous antibiotics were used, followed by return to CIC. On 2026-02-10, she underwent transurethral cystoscopic lithotripsy for bladder stones. At the final assessment (age 1 year and 3 months), lower limb proximal strength improved to grade 4/5, but distal strength was grade 2/5, with bilateral foot drop (left more prominent). Sensory examination showed questionable perineal sensation; the patient was poorly cooperative. Bowel function showed constipation without fecal incontinence, requiring lactulose and glycerin enema. Anal reflex was normal. Bilateral pathological reflexes were consistently negative. Somatosensory evoked potentials showed normal bilateral ankle-cortex and wrist-cortex pathways; right femoral nerve CMAP amplitude improved compared with 2025-08-13, bilateral tibial nerve CMAP amplitude decreased, and right common peroneal nerve CMAP was unchanged. She remained with a neurogenic bladder requiring CIC. No further febrile urinary tract infections occurred after stone removal. Long-term prognosis depends on persistent neurogenic bladder; lifelong urological follow-up, CIC, renal ultrasound, and monitoring for stones or recurrent UTI are required.

## Discussion

This case highlights three clinical lessons: the diagnostic significance of a prolonged febrile prodrome, the risk of sequential polymicrobial superinfection in the neurogenic bladder, and the need for prolonged, susceptibility-guided antimicrobial therapy with source control. Importantly, several positive urine cultures represented colonization rather than true infection (15), and the emergence of fluconazole resistance may have been promoted by treating colonization (16, 17). This case therefore serves as a cautionary antimicrobial-stewardship narrative.

First, the patient's 4-week intermittent fever without neurological signs initially obscured the spinal pathology. This prodrome likely reflected intermittent bacterial seeding of the epidermoid cyst or subclinical inflammation along the sinus tract. When neurological deficits emerged, they were catastrophic (18). This pattern is consistent with previous reports that diagnostic delays are common in infants, with many already having deficits at presentation (19). Unexplained prolonged fever in an infant should therefore prompt careful examination for sacral dimples or hypertrichosis (20).

Second, the sequential shift from Klebsiella aerogenes to C. glabrata and then E. faecium illustrates the microbiological challenge of recurrent complicated UTI in a neurogenic bladder (21). Biofilm formation on bladder stones and urothelium likely provided a protective reservoir (22). The acquisition of fluconazole resistance in C. glabrata emphasizes the need for repeated susceptibility testing (23). However, it is also possible that this resistance emerged because asymptomatic candiduria was treated. IDSA 2016 recommends against treating asymptomatic candiduria except in neutropenia, very-low-birth-weight infants, or before urologic manipulation (24). In our patient, the C. glabrata count of 6.8 × 10^4^ CFU/mL was below the usual significance threshold (25), and treatment was based on pyuria and urinary sediment. Prolonged broad-spectrum antibiotics likely contributed to fungal selection (26). Linezolid was used earlier for a linezolid-susceptible Staphylococcus hominis CVC tip culture and was discontinued before the E. faecium isolate appeared; the latter was considered colonization. Recurrent colonization was distinguished from true infection by combining colony counts, pyuria, fever or clinical symptoms, elevated inflammatory markers, and repeated cultures (27).

Third, rescue required a multifaceted approach rather than short-course therapy. The regimen included targeted bactericidal therapy, echinocandin-based therapy for resistant candiduria, prolonged combination therapy for enterococcal eradication, and suppressive therapy plus source control. Although micafungin has poor urinary excretion, it was selected because the C. glabrata isolate was fluconazole-resistant and amphotericin B was avoided because of nephrotoxicity. Echinocandins achieve therapeutic concentrations in bladder tissue and renal parenchyma, have activity against Candida biofilms, and have been reported in case series of candiduria (28). Clearance likely reflected a combination of micafungin, repeated culture-guided therapy, CIC optimization, and stone removal; we cannot attribute success to micafungin alone. This complex regimen should not be considered a “blueprint” but rather a cautionary example of how treating colonization can drive resistance (29). Previous reports of infantile CDS with infected epidermoid cysts have mainly focused on acute neurological presentation and surgical outcomes. Ackerman and Menezes reported a 30-year operative series of 28 patients, including only one infant, without long-term urological or microbiological follow-up. Acharyya (3) et al. reported an 11-month-old infant with CDS and an infected spinal epidermoid cyst who had recurrent overflow incontinence, three documented UTIs in 4 months, a multiresistant E. coli urine culture, and a Gram-negative bacillus from the cyst resembling the urine isolate; bladder dysfunction resolved with no further UTI. However, serial susceptibility data and longitudinal microbiological follow-up were not detailed (4). In contrast, our case provides serial urine cultures, susceptibility data, and a source-control-based salvage strategy, adding to the limited literature on post-CDS neurogenic bladder and recurrent complicated UTI.

Finally, the MRI findings were consistent with CDS complicated by an infected spinal epidermoid cyst. Whole-spine MRI showed diffuse spinal cord swelling and signal abnormality. Contrast-enhanced MRI revealed a linear sacral tract communicating with the spinal canal and an intradural cystic lesion at L1–S2. Follow-up MRI showed T2 heterogeneous hyperintensity, T1 hypointensity, local diffusion restriction, and rim enhancement, consistent with residual/recurrent infection. Serial renal and bladder ultrasound was essential for detecting bladder stones and hydronephrosis. Imaging studies were performed at an outside institution; original images were not available for publication. The radiological findings described here are based on outside imaging reports and clinical records.

## Limitations

This study has several limitations. First, it is a single case and may not be generalizable. Second, although urine sterilization was achieved, long-term follow-up is needed to assess recurrent UTI and hydronephrosis. Third, urodynamic studies were not performed because of the patient's age, the need for sedation, and the lack of standardized normative pediatric data; bladder management was guided by ultrasound, residual urine, and clinical symptoms. Fourth, the dura was not actively opened, but a localized dural defect was present intraoperatively, and no CSF leak occurred. Fifth, bladder stone analysis was not performed, limiting etiological inference. Sixth, urinalysis and clinical symptom data were incomplete for some episodes, and specimen collection methods varied. Seventh, we cannot definitively establish causality between UTI and bladder stones. Eighth, all MRI and other imaging studies were performed at an outside institution, and original images could not be retrieved for de-identified publication; the radiological descriptions are based on outside imaging reports and clinical records.

## Conclusions

The management of CDS with secondary infected epidermoid cysts extends beyond neurosurgical resection. This case provides three lessons: (1) prolonged febrile prodrome without neurological signs should prompt evaluation for cutaneous stigmata of spinal dysraphism; (2) postoperative neurogenic bladder in infants creates a high-risk environment for recurrent complicated UTI with sequential bacterial and fungal superinfections, including azole resistance; and (3) eradication requires a prolonged, multidisciplinary, susceptibility-guided approach with definitive source control. This report adds to the limited literature by documenting detailed longitudinal microbiological follow-up after CDS surgery. The emergence of fluconazole resistance in C. glabrata may have been promoted by treating colonization, highlighting the need for strict UTI definitions in catheterized infants. Long-term prognosis depends on persistent neurogenic bladder; lifelong urological follow-up, CIC, renal ultrasound, and monitoring for stones or recurrent UTI are required. No febrile UTI recurred after stone removal at the final follow-up.

## Data Availability

The original contributions presented in the study are included in the article/Supplementary Material, further inquiries can be directed to the corresponding author.

## References

[1] Vokshoor A Jajj H Grunwald T Kolker S Petros J. Posterior cervical congenital dermal sinus tract: case report and review of literature. Spinal Cord Ser Cases. (2023) 9(1):40. 10.1038/s41394-023-00575-5 37532714 PMC10397304

[2] Gao G Chen Y Tao B Sun M Bai S Shang A. Early surgical intervention with antibiotic treatment for congenital dermal sinus with central nervous system infection: a retrospective study of 20 cases. World Neurosurg. (2022) 164:e17–23. 10.1016/j.wneu.2022.02.102 35247614

[3] Acharyya S Chakravarty S. Congenital dermal sinus with extensive intramedullary expansion and an infected spinal epidermoid cyst in an infant. J Family Med Prim Care. (2018) 7(5):1103–5. 10.4103/jfmpc.jfmpc_158_18 30598968 PMC6259516

[4] Gupta SK Singh P Gupta RK Sharma R Nehete LS. Infected congenital lumbosacral dermal sinus tract with conus epidermoid abscess: a rare entity. Childs Nerv Syst. (2021) 37(3):741–7. 10.1007/s00381-020-04987-8 33247382

[5] De Gennaro M Lais A Fariello G Caldarelli M Capozza N Talamo M. Early diagnosis and treatment of spinal dysraphism to prevent urinary incontinence. Eur Urol. (1991) 20(2):140–5. 10.1159/000471684 1752272

[6] Mariani F Ausili E Zona M Grotti G Curatola A Gatto A. The impact of constant antibiotic prophylaxis in children affected by spinal dysraphism performing clean intermittent catheterization: a 2-year monocentric retrospective analysis. Childs Nerv Syst. (2022) 38(3):605–10. 10.1007/s00381-021-05337-y 34523011 PMC8917099

[7] Ben-David R Carroll F Kornitzer E Dekalo S Mano R Ben-Chaim J. Asymptomatic bacteriuria and antibiotic resistance profile in children with neurogenic bladder who require clean intermittent catheterization. Spinal Cord. (2022) 60(3):256–60. 10.1038/s41393-021-00679-5 34446838

[8] Linsenmeyer TA. Catheter-associated urinary tract infections in persons with neurogenic bladders. J Spinal Cord Med. (2018) 41(2):132–41. 10.1080/10790268.2017.1415419 29323625 PMC5901448

[9] Bjerklund Johansen TE Bahrs C Bruyere F Cai T Joseph A Köves B. Consensus position statement on advancing the classification of patients and tests of cure in studies of antibiotic treatment of complicated urinary tract infections. Lancet Infect Dis. (2025) 25(10):e605–18. 10.1016/S1473-3099(25)00142-2 40378860

[10] Trautner BW Cortés-Penfield NW Gupta K Hirsch EB Horstman M Moran GJ. Clinical practice guidelines by infectious diseases society of America (IDSA): 2025 guidelines on management and treatment of complicated urinary tract infections–duration of antibiotics for complicated UTI. Clin Infect Dis. (2026) 82(Suppl 3):i79–88. 10.1093/cid/ciaf462 41419448

[11] Erdoğan E Yetişgen A Doğan S Sinanoğlu MS Kılıç FE Kurt O. Multidrug-resistant uropathogens in pediatric urinary tract infections: a multicenter retrospective trend analysis (2020-2024). Naunyn Schmiedebergs Arch Pharmacol. (2026) 399(8):11827–43. 10.1007/s00210-026-05134-x PMC13269392 41735705

[12] El Zein Z Boutros CF El Masri M El Tawil E Sraj M Salameh Y. The challenge of multidrug resistance in hospitalized pediatric patients with urinary tract infections. Front Cell Infect Microbiol. (2025) 15:1570405. 10.3389/fcimb.2025.1570405 40667421 PMC12259619

[13] Zhou G Jiang M Liu X Li S Chen J. Risk factors associated with recurrent febrile urinary tract infection in children with neurogenic bladder who perform clean intermittent catheterization. Neurourol Urodyn. (2023) 42(7):1485–90. 10.1002/nau.25245 37421159

[14] Madden-Fuentes RJ McNamara ER Lloyd JC Wiener JS Routh JC Seed PC. Variation in definitions of urinary tract infections in spina bifida patients: a systematic review. Pediatrics. (2013) 132(1):132–9. 10.1542/peds.2013-0557 23796735

[15] Schartz WR Bennett N Aragon L Kennedy K Wilson A Boyd S. Templated microbiology comments with candiduria to enhance antimicrobial stewardship. Antimicrob Steward Healthc Epidemiol. (2022) 2(1):e156. 10.1017/ash.2022.292 36483356 PMC9726485

[16] Zeitoun H Salem RA El-Guink NM Tolba NS Mohamed NM. Elucidation of the mechanisms of fluconazole resistance and repurposing treatment options against urinary Candida spp. isolated from hospitalized patients in Alexandria, Egypt. BMC Microbiol. (2024) 24(1):383. 10.1186/s12866-024-03512-0 39354378 PMC11443771

[17] ElKenawy RH Zaki MES Essa SG Mahmoud NM El-Nagdy AH Abdellatif M. Molecular study of candiduria in pediatric patients in in relation to biofilm formation and fluconazole tolerance. Diagn Microbiol Infect Dis. (2025) 113(2):116943. 10.1016/j.diagmicrobio.2025.116943 40505521

[18] Dho YS Kim SK Wang KC Phi JH. Catastrophic intramedullary abscess caused by a missed congenital dermal sinus. J Korean Neurosurg Soc. (2015) 57(3):225–8. 10.3340/jkns.2015.57.3.225 25810867 PMC4373056

[19] Li D Zheng S Xiao X Ma Y Zhang Q Dong C. Clinical characteristics and surgical treatment of congenital gluteal dermal sinus tract in children: a 15-year retrospective single-center clinical experience. Front Pediatr. (2026) 14:1836614. 10.3389/fped.2026.1836614 42181559 PMC13194606

[20] Stresemann S Westrick M Fitch T Amin O. Recurrent bacterial meningitis in an infant caused by a sacral dermal sinus tract. Cureus. (2026) 18(4):e107220. 10.7759/cureus.107220 42153061 PMC13180298

[21] Andretta E Longo R Balladelli M Sgarabotto C Sgarabotto D. Intravesical gentamicin: an option for therapy and prophylaxis against recurrent UTIs and resistant bacteria in neurogenic bladder patients on intermittent catheterization. Antibiotics. (2022) 11(10):1335. 10.3390/antibiotics11101335 36289993 PMC9598998

[22] Yu Y Singh H Tsitrin T Bekele S Lin YH Sikorski P. Urethral catheter biofilms reveal plasticity in bacterial composition and metabolism and withstand host immune defenses in hypoxic environment. Front Med. (2021) 8:667462. 10.3389/fmed.2021.667462 PMC8260951 34249966

[23] Pfaller MA Castanheira M Lockhart SR Ahlquist AM Messer SA Jones RN. Frequency of decreased susceptibility and resistance to echinocandins among fluconazole-resistant bloodstream isolates of Candida glabrata. J Clin Microbiol. (2012) 50(4):1199–203. 10.1128/JCM.06112-11 22278842 PMC3318516

[24] Pappas PG Kauffman CA Andes DR Clancy CJ Marr KA Ostrosky-Zeichner L. Clinical practice guideline for the management of candidiasis: 2016 update by the infectious diseases society of America. Clin Infect Dis. (2016) 62(4):e1–50. 10.1093/cid/civ933 26679628 PMC4725385

[25] Leis JA Rebick GW Daneman N Gold WL Poutanen SM Lo P. Reducing antimicrobial therapy for asymptomatic bacteriuria among noncatheterized inpatients: a proof-of-concept study. Clin Infect Dis. (2014) 58(7):980–3. 10.1093/cid/ciu010 24577290

[26] Thomas-Rüddel DO Schlattmann P Pletz M Kurzai O Bloos F. Risk factors for invasive Candida infection in critically ill patients: a systematic review and meta-analysis. Chest. (2022) 161(2):345–55. 10.1016/j.chest.2021.08.081 PMC8941622 34673022

[27] Forster CS Jackson E Ma Q Bennett M Shah SS Goldstein SL. Predictive ability of NGAL in identifying urinary tract infection in children with neurogenic bladders. Pediatr Nephrol. (2018) 33(8):1365–74. 10.1007/s00467-018-3936-0 29532235 PMC6218164

[28] Javorova Rihova Z Slobodova L Hrabovska A. Micafungin is an efficient treatment of multi drug-resistant Candida glabrata urosepsis: a case report. J Fungi. (2021) 7(10):800. 10.3390/jof7100800 PMC8538929 34682222

[29] Fan X Tsui CKM Chen X Wang P Liu ZJ Yang CX. High prevalence of fluconazole resistant Candida tropicalis among candiduria samples in China: an ignored matter of concern. Front Microbiol. (2023) 14:1125241. 10.3389/fmicb.2023.1125241 36937265 PMC10017723

